# A case of recalcitrant acute generalized exanthematous pustulosis with Sjogren's syndrome: Successfully treated with low‐dose cyclosporine

**DOI:** 10.1002/ccr3.2352

**Published:** 2019-08-04

**Authors:** Zeynep Gizem Kaya İslamoğlu, Pınar Karabağli

**Affiliations:** ^1^ Department of Dermatology, Faculty of Medicine Selcuk University Konya Turkey; ^2^ Department of Pathology, Faculty of Medicine Selcuk University Konya Turkey

**Keywords:** acute generalized exanthematous pustulosis, cyclosporine, hydroxychloroquine, Sjogren's syndrome

## Abstract

Acute generalized exanthematous pustulosis (AGEP) is a reaction attributed mainly to drugs. Hydroxychloroquine is a rare cause of AGEP especially used in rheumatology and dermatology. Systemic corticosteroids are the first‐line treatment agents in AGEP. But cyclosporine can be a good choice for patients resistant to systemic corticosteroid treatment in AGEP.

## 1 INTRODUCTION

Acute generalized exanthematous pustulosis (AGEP) is a pustular severe cutaneous adverse reaction usually based on the drugs. We report a 64‐year‐old woman who developed AGEP after a 20‐day course of hydroxychloroquine and might be treated with cyclosporine because of unresponsiveness to the first‐line treatments.

Severe cutaneous adverse reactions to drugs are associated with morbidity, mortality, healthcare costs, and drug development challenges. They are a wide spectrum of entities consisting of Stevens‐Johnson syndrome, toxic epidermal necrolysis, drug reaction with eosinophilia and systemic symptoms syndrome, and acute generalized exanthematous pustulosis (AGEP).^1^ The term of AGEP is first used in 1980 by Beylot et al in the medical literature as a separate entity.^2^ It is a rare adverse drug reaction represented by suddenly onset of widespread nonfollicular sterile pustules on erythematous and edematous plaques, high fever (>38°C), and neutrophilia (>5000/mL). The initial lesions start from the face and fold areas and rapidly dissipate. Targetoid lesions and purpura can be observed in some patients. Mucosal involvement is uncommon. The reaction resolves fully in 15 days.^3^ The reported mortality is 5%.^4^

Many drugs have been associated with AGEP, such as ampicillin/amoxicillin, quinolones, sulfonamides, diltiazem, antimalarials, and terbinafine.^5^ Suspicious drug cessation is usually sufficient with superficial desquamation following a rapid recovery in 1‐2 weeks. If necessary, systemic corticosteroids can be used in severe cases to hasten resolution.^3^ Here, we reported a case of AGEP induced by hydroxychloroquine (HCQ) who was resistant to high doses of systemic corticosteroids that cleared successfully after treatment with low dose of cyclosporine (CsA).

## 2 CASE REPORT

**Figure 1:**
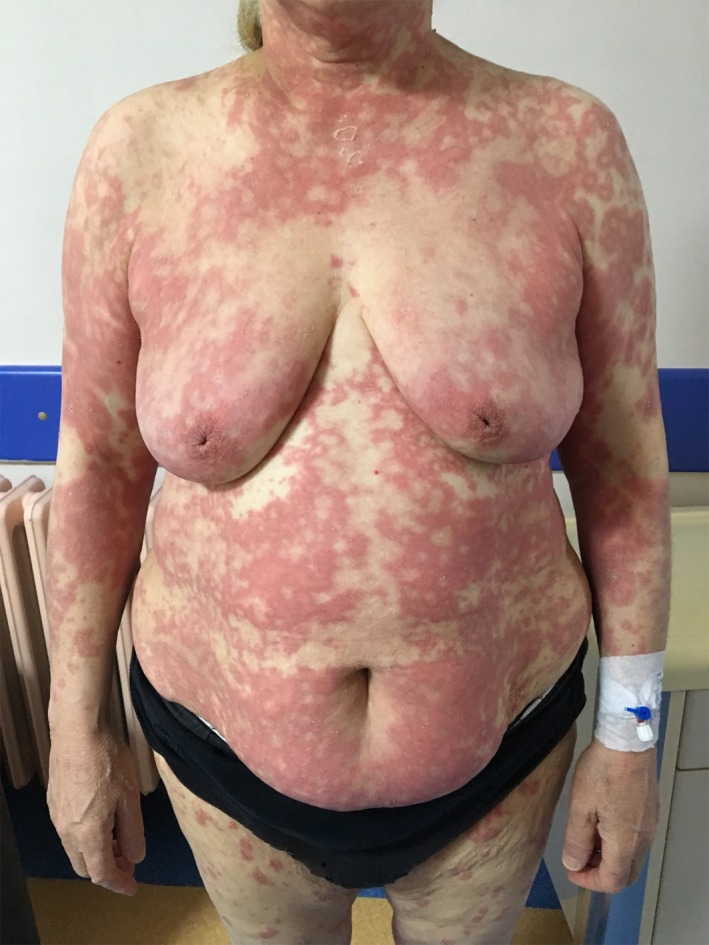
Diffuse erythematous and edematous plaques with nonfollicular pustules on her body

**Figure 2:**
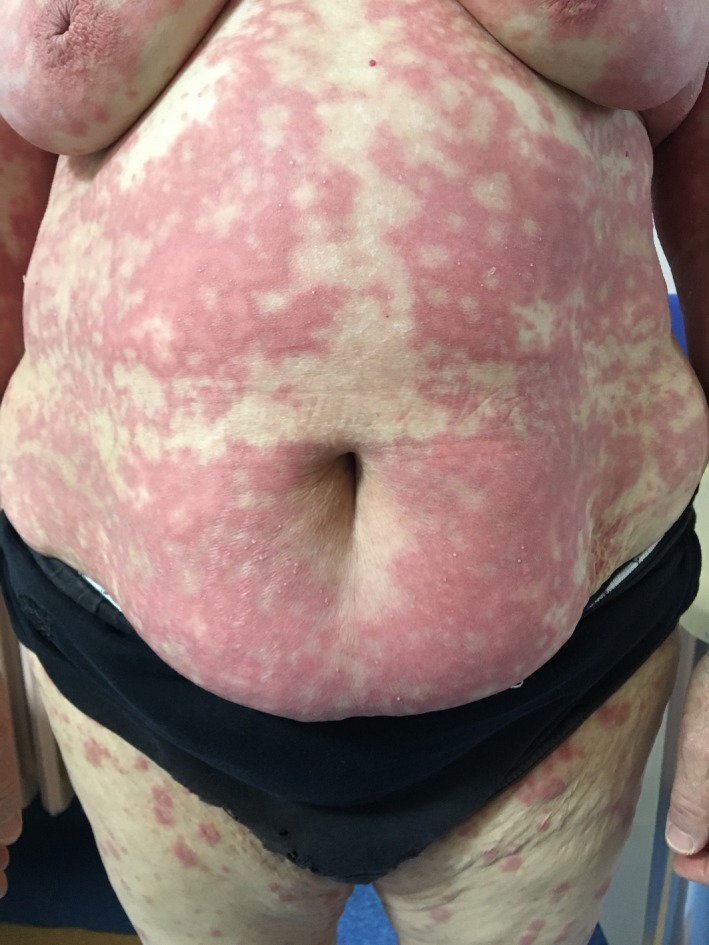
A closer screenshot of plauqes

**Figure 3:**
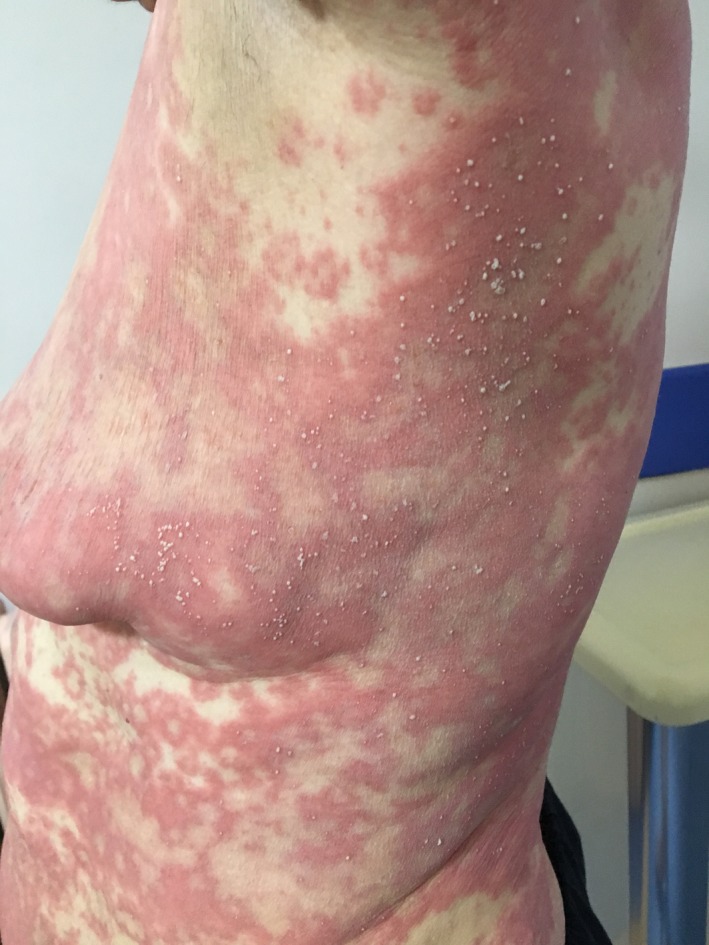
Nonfollicular pustules

**Figure 4:**
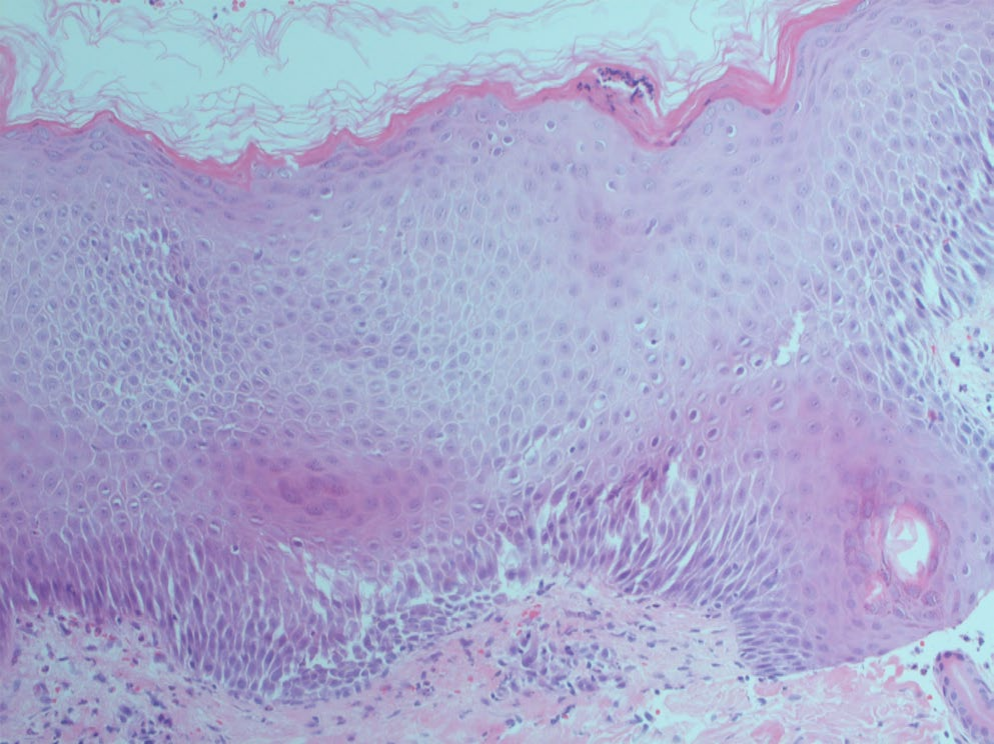
HEX20: Corneal/subcorneal pustules, edema, spongious, and perivascular mixed lymphocytic infiltrate with multiple neutrophils, a few eosinophils

**Figure 5:**
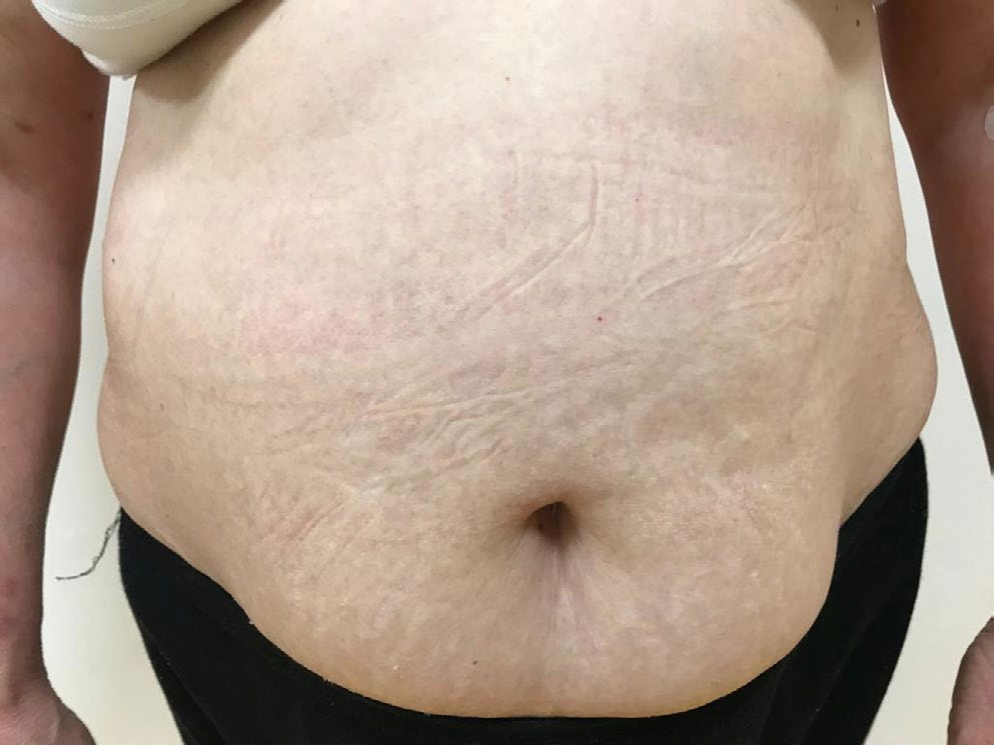
Total clearance after 22 d of cyclosprin

A 64‐year‐old woman with a history of HCQ use initially noted the appearance of pustules on her axillary fold and inguinal area. Over the following days, she came with widespread pustules on all her body. The rash was started twenty days after starting HCQ. She had no additional drug use out of HCQ. She had a past medical history of Sjogren's syndrome and allergic asthma. Dermatological examination showed itchy, diffuse erythematous, and edematous plaques with nonfollicular pustules on her extremities and body and minimal facial edema (Figures 1, 2, 3). Oral mucosa and genital mucosa were normal. She had no fever. Laboratory evaluation showed leukocytosis of 18.3 K/uL with absolute neutrophil count of 15.2 K/uL. Liver and renal function testing was normal. There was no reproduction in the culture of pustule. We took a biopsy for differentiating AGEP from pustular psoriasis. Histopathologic examination revealed corneal/subcorneal pustules, edema, spongious, and perivascular mixed lymphocytic infiltrate with multiple neutrophils, a few eosinophils (Figure 4). The patient was diagnosed with AGEP according to the clinical, laboratory, and histopathologic findings. Differences in localization and spongiform character of pustules in the biopsy can provide a hint for differentiating AGEP from pustular psoriasis. Also, there were no features of plaque‐type psoriasis in the histopathology and eosinophils were seen which suggests a hypersensitivity reaction. HCQ was immediately withdrawn on admission. At first, she was treated with low‐dose systemic corticosteroids (40 mg/d of methylprednisolone), antihistamines, and topical emollients. When the patient did not respond to treatment within a few days, the dose of corticosteroid was gradually increased to 120 mg/d. She had taken these treatments about 15 days. Given the lack of efficacy, methylprednisolone was discontinued and cyclosporine was started (2.5 mg/kg/d). After 13 days, the previously existing lesions had resolved and also new lesion outbreaks stopped. Within 22 days, she was in clinical remission on CsA therapy and had no relapses and side effects during the treatment (Figure 5). After that, the dose of CsA was reduced in a short time (About 15 days). The patient remains in complete clinical remission and free of disease for a 3‐month follow‐up.

## 3 DISCUSSION

Hydroxychloroquine is an antimalarial drug with immunosuppressive and anti‐inflammatory properties. It is commonly used in rheumatic and dermatological disorders. It has been reported as a rare cause of AGEP.^6^ A total of twenty‐one cases of AGEP with HCQ following a PubMed search can be found in the literature.^7^ And, there are three cases reported as corticosteroid‐refractory AGEP induced by HCQ successfully treated with CsA.^8^, ^9^, ^10^

There are two different latency periods for AGEP in case‐control studies, that is, 1 day for antibiotics and 11 days with a range of 4‐30 days for nonantibiotics including HCQ. HCQ‐induced AGEP cases are generally recalcitrant to standard treatments and need a longer duration. This can be explained by the lengthy half‐life of HCQ approximately 40‐50 days.^3^

The first mandatory steps of AGEP are culprit‐drug identification and its early withdrawal in all patients. Corticosteroids are not always administered in most cases. CsA is not the first‐line therapy for AGEP but it was used for cases which were recalcitrant to corticosteroid therapy in the literature.^8^, ^9^, ^10^ Table 1 summarizes previously reported three cases of AGEP with HCQ recalcitrant to corticosteroid therapy. In present case, we gave CsA at lower doses and in shorter periods than the reported cases.

**Table 1:** Summary of patients treated with CsA for AGEP

	Age‐Gender	Underlying rheumatologic disease	Days of illness before CsA initiated	Initial dose of CsA	Days of CsA therapy before clearance
Di Lernia et al (2009)^8^	63‐Female	Rheumatoid arthritis	25	5 mg/kg	30
Yalçın et al (2015)^9^	67‐Female	Seronegative polyarthritis	22	4 mg/kg	–
Castner et al (2017)^10^	56‐Female	Rheumatoid arthritis	7	4.5 mg/kg	10

In conclusion, AGEP due to HCQ is a rare adverse drug reaction and they are generally severe and recalcitrant. We can usually link this to the immunologic dysregulation due to an underlying rheumatologic disease. In contrast to other three reported cases, Sjogren's syndrome was prominent in our case. To our knowledge, this is the fourth case of AGEP successfully treated with cyclosporine. Cyclosporine has many inhibitory effects on T cells. And in AGEP, T cells play an important role in the pathogenesis. For this reason, CsA is effective in the treatment of recalcitrant AGEP. In our option, CsA can be a good choice for patients resistant to systemic corticosteroid treatment. Even at the lowest doses and in short periods of time can provide us with charming results.

## CONFLICT OF INTEREST

None declared.

## AUTHOR CONTRIBUTIONS

ZGKİ: has been the corresponding author, has made the design of the case and collected the data, and has been involved in idea, drafting, writing the manuscript, and revising it critically. PK: has been involved in collecting and editing the histopathological data and preparation of the manuscript. All the authors approved the final version of the case report for submission to the Clinical Case Reports.
